# A cross-sectional survey on mother-to-child transmission of HIV among the migrant population in Dongguan, China

**DOI:** 10.3389/fgwh.2023.1106959

**Published:** 2023-10-06

**Authors:** Congcong Pan, Haiyan Pan, Dongmei Liang, Yuanyuan Liu, Sichun Yin, Jianbo Zhong, Songmei He

**Affiliations:** ^1^ School of Public Health, Guangdong Medical University, Dongguan, China; ^2^ Department of Infectious Diseases, Dongguan People’s Hospital, Dongguan, China

**Keywords:** migrant population, HIV vulnerability, prevention strategies, mother-to-child transmission, sociodemographic factors, antiretroviral therapy

## Abstract

**Introduction:**

The migrant population, consisting of individuals who relocate from rural to urban areas, faces unique challenges that heighten their vulnerability to HIV infection. These challenges stem from a combination of sociodemographic factors and limited access to healthcare services. Understanding the dynamics of HIV transmission within this population is crucial for the development of effective prevention strategies.

**Methods:**

To investigate the factors contributing to HIV vulnerability among migrants, we conducted a cross-sectional study at Dongguan People's Hospital from January 1, 2018, to December 31, 2021. Our study focused on pregnant women living with HIV and their infants, with a particular emphasis on sociodemographic characteristics, HIV testing and treatment profiles, and neonatal clinical data. Data were systematically collected using standardized forms.

**Results:**

Analysis of data from 98 participants revealed noteworthy findings. No significant associations were observed between age, marital status, and educational background regarding HIV vulnerability. Similarly, factors such as the status of sexual partners, spousal therapy, and the number of children had no significant impact. However, our analysis highlighted the critical role of treatment strategies for HIV-positive women and the timing of antiretroviral therapy initiation for women with HIV, both of which were associated with HIV transmission (*P* < 0.05). Additionally, factors such as feeding type, neonatal antiretroviral prophylaxis, and preventive treatment strategies showed significant associations, while the preventive treatment program for neonates demonstrated no significant impact.

**Discussion:**

These findings provide valuable insights into the specific risk factors and barriers to HIV prevention faced by the migrant population in Dongguan. They underscore the importance of targeted interventions and policies aimed at curtailing mother-to-child HIV transmission. By addressing the unique challenges experienced by migrant mothers and their infants, this study contributes significantly to broader efforts in controlling the spread of HIV, ultimately enhancing the health outcomes and well-being of Dongguan's migrant population. Furthermore, our research introduces a distinctive perspective within the extensively examined domain of Prevention of Mother-to-Child Transmission (PMTCT) programs, focusing on the internally migrant Chinese population, an understudied demographic group in this context. This study, conducted in Dongguan, China, represents one of the pioneering investigations into pregnant women with HIV and their infants within this migrant community.

## 1. Introduction

The prevention of mother-to-child transmission (PMTCT) of HIV is a global health priority, as the HIV epidemic has significant implications for the health of women and neonates, as well as socioeconomic development (1). Over the past 20 years, PMTCT has been recognized as one of the most successful public health interventions. In China, PMTCT of HIV infection has emerged as a critical public health priority since 2002 (2). The intervention program ensures that every pregnant woman attending antenatal care (ANC) clinics receives free HIV counseling and testing. HIV-positive pregnant women are then referred to specialized hospitals for further examination and medication, including the provision of free antiretroviral drugs. Additionally, neonates born to HIV-positive mothers receive free antiretroviral medication within 6–12 h after birth. National guidelines recommend early diagnosis and HIV testing at the sixth week and third month (3). These comprehensive measures have contributed to significant progress in preventing mother-to-child HIV transmission in China (4).

Recent amendments to China's family planning policies have resulted in increased demand for a third child among individuals living with HIV. Early diagnosis of neonatal HIV infection is a crucial component of preventing mother-to-child transmission, and it should be carried out effectively and efficiently in China. However, access to PMTCT services among the migrant population in China remains limited, posing a challenge in the fight against HIV. Migrants are individuals who have left their original residences and now live in different areas (5). In various countries, including China, migrants are considered a high-risk group for HIV transmission. Studies conducted by the Centers for Disease Control and Prevention in the United States in 1987 and 1992 reported HIV infection rates of 2.6% to 5% among migrant populations in urban communities, which is ten times higher than the general population (6). In China, more than two-thirds of HIV infections detected in urban areas such as Shanghai, Shanxi, and Zhejiang between 1995 and 2000 were among immigrants (7–9). As of 2021, China has a migrant population of 376 million, with an average age of 29.8, which is associated with a higher risk of HIV infection and transmission (10, 11). Insufficient prenatal care among migrant women living with HIV is one of the pressing problems in PMTCT implementation. Reports indicate that only about 60% of migrant women living with HIV receive prophylaxis with antiretroviral therapy, approximately 33% of HIV-exposed neonates do not receive HIV tests, and 22% do not receive prophylaxis with antiretroviral therapy (12, 13).

Migrant populations are vulnerable to various health risks, including infectious diseases, occupational diseases, reproductive health issues, and mental health problems, due to their harsh working and living environments, as well as the lack of social security and support (14). Dongguan, ranking 10th among migrant cities in China, has a permanent population of 10.4666 million, of which the migrant population is 7.9522 million (15). The city's well-developed transportation system and thriving economy have attracted a large influx of young people, contributing to the rapid spread of HIV. Data from the seventh national census reveals that individuals aged 15–59 in Dongguan account for 81.41% of the population, with an average age of approximately 34 years, similar to the city's overall age distribution. In 2021, 90% of HIV-positive patients tested in Dongguan were from the migrant population, making case follow-up challenging. Furthermore, Dongguan has the highest birth rate among cities in Guangdong Province in 2021, further increasing the risk of mother-to-child transmission of HIV within the migrant population.

The risk of mother-to-child transmission of HIV among the migrant population in Dongguan is on the rise. Although the Chinese government has issued guidelines for comprehensive PMTCT work, there remains a significant gap between policy and actual practice (16). Achieving the goal of eliminating mother-to-child HIV transmission proposed by the World Health Organization requires further efforts in improving the implementation of PMTCT and bridging existing gaps.

## 2. Methods

### 2.1. Study design

This cross-sectional study aimed to investigate the mother-to-child transmission of HIV among pregnant women and their babies hospitalized at Dongguan People's Hospital between January 1, 2018, and December 31, 2021. All participants in this study were migrants, defined as individuals or groups of people who relocate from one geographical area to another, including movement from rural to urban areas or between different regions. The study acknowledges the diverse nature of migration patterns and its implications for public health and social dynamics.

### 2.2. Participants

The study included a sample of 98 pregnant women living with HIV and their neonates (≤12 months) who received an HIV infant prophylaxis regimen at Dongguan People's Hospital during the specified study period. The participants were selected based on their HIV-positive status and the availability of neonates for follow-up. All participants were migrants. Non-Chinese migrant women were excluded from this study.

### 2.3. Data collection

Data were collected using standardized data collection forms. Sociodemographic data of the pregnant women, including age, marital status, educational background, and migration status, were recorded. Information on HIV testing and treatment, such as the status of sexual partners, spousal therapy, antiretroviral therapy, and mode of delivery, was also documented. Clinical data of the neonates, such as gender, number of children, and feeding method, were collected. Ethical approval for the study was obtained from Dongguan People's Hospital.

### 2.4. Data analysis

The collected data were subjected to descriptive, correlational, and bivariate regression analyses. Categorical data were presented as component proportions (%). The constituent ratios between two or more groups were compared using statistical tests such as the chi-square or Fisher's exact test. A significance level of *P* < 0.05 was considered statistically significant. The statistical analysis was performed using SPSS version 24.0 (IBM Corporation, Armonk, NY, USA).

## 3. Results

### 3.1. Distribution of characteristics among participants

The distribution of age among the participants revealed that 31.6% were aged 20–29, 62.2% were aged 30–39, and 6.2% were aged 40 or above. The majority of participants were married (98%) and had completed junior high school education (85.7%). Among the participants, 32.7% had a sexual partner who tested negative for HIV, while 67.3% had a positive sexual partner. Spousal therapy and antiretroviral therapy were received by 32.7% of the participants, while 67.3% did not receive such treatment. Regarding the number of children, 65.3% had one child, 27.5% had two children, and 7.2% had three children. The gender distribution of the children born to the participants was 48% male, 38.7% female, and 13.3% unclear. When asked about having a baby after testing positive for HIV, 34.7% responded affirmatively, while 65.3% did not. Finally, 99% of the participants expressed a desire. Detailed information can be found in Table 1.

**Table 1:** Demographic characteristics of women living with HIV (*n* = 98).

Variable	*n*	%
Age		
20–29	31	31.6
30–39	61	62.2
≥40	6	6.2
Population classification		
Household population	47	48
Migrant population	51	52
Marital status		
Unmarried	2	2
Married	96	98
Educational background		
Junior high school	84	85.7
Senior high school	14	14.3
Status of sexual partner		
Negative	32	32.7
Positive	66	67.3
Spousal therapy and antiretroviral therapy		
Yes	32	32.7
No	66	67.3
Number of children		
1	64	65.3
2	27	27.5
3	7	7.2
Gender of child		
Male	47	48
Female	38	38.7
Unclear	13	13.3
Whether to have a baby after a positive test		
Yes	34	34.7
No	64	65.3
Planning for another pregnancy		
Yes	97	99
No	1	1

### 3.2. Characteristics of positive neonatal mothers

Among the positive neonatal mothers, the majority were between the ages of 20 and 39, with 30% (*n* = 3) aged 20–29% and 70% (*n* = 7) aged 30–39. All positive neonatal mothers were unmarried (100%, *n* = 10) and had completed junior high school education (100%, *n* = 10). Half of the positive neonatal mothers had negative sexual partners (50%, *n* = 5), while the other half had positive partners (50%, *n* = 5). Among these mothers, 40% (*n* = 4) received spousal therapy and antiretroviral therapy, while 60% (*n* = 6) did not. In terms of the number of children, 80% (*n* = 8) had one child, 10% (*n* = 1) had two children, and 10% (*n* = 1) had three children. The gender distribution of the children born to positive neonatal mothers was 60% (*n* = 6) males, 30% (*n* = 3) females, and 10% (*n* = 1) cases where the gender was unclear. The majority of positive neonatal mothers intended to have a baby after a positive test (90%, *n* = 9), while a small proportion did not (10%, *n* = 1). Additionally, 60% (*n* = 6) of the positive neonatal mothers planned for another pregnancy, while 40% (*n* = 4) did not. Detailed information can be found in Table 2.

**Table 2:** Demographic characteristics of positive neonatal mothers (*n* = 10).

Variable	*n*	%
Age		
20–29	3	30
30–39	7	70
Marital status		
Unmarried	10	100
Married	0	0
Educational background		
Junior high school	10	100
Senior high school	0	0
Status of sexual partner		
Negative	5	50
Positive	5	50
Spousal therapy and antiretroviral therapy		
Yes	4	40
No	6	60
Number of children		
1	8	80
2	1	10
3	1	10
Gender of child		
Male	6	60
Female	3	30
Unclear	1	10
Whether to have a baby after a positive test		
Yes	9	90
No	1	10
Planning for another pregnancy		
Yes	6	60
No	4	40

### 3.3. Characteristics of antiretroviral treatment among pregnant women and neonates

Statistical analysis of the antiretroviral treatment among pregnant women living with HIV and neonates revealed that 66.3% (*n* = 65) of women received any form of antiretroviral therapy, while 33.7% (*n* = 33) did not. Among the women receiving antiretroviral therapy, 2.0% (*n* = 2) started treatment before pregnancy, 59.2% (*n* = 58) started during pregnancy, 18.4% (*n* = 18) started at or after delivery, and 20.4% (*n* = 20) had an unclear starting point. The most common treatment strategies used for positive women were lamivudine/zidovudine/lopinavir-ritonavir (21.4%, *n* = 21) and lamivudine/tenofovir/lopinavir-ritonavir (28.6%, *n* = 28). Other treatment strategies were used by 12.2% (*n* = 12) of women, and 37.8% (*n* = 37) had an unclear treatment strategy. Regarding the mode of delivery, 35.8% (*n* = 35) of women had a vaginal delivery, 61.2% (*n* = 60) underwent a cesarean section, and 3.1% (*n* = 3) had an unclear type of delivery. The majority of neonates were formula-fed (94.9%, *n* = 93), while 6.1% (*n* = 6) were breastfed. Antiretroviral prophylaxis was given to 85.7% (*n* = 84) of neonates, with 14.3% (*n* = 14) not receiving prophylaxis. Among neonates receiving preventive treatment, 14.3% (*n* = 14) received treatment within 28 days, 10.2% (*n* = 10) between 29 and 42 days, 58.2% (*n* = 57) beyond 42 days, and 17.3% (*n* = 17) had an unclear duration of treatment. The majority of neonates received monotherapy for preventive treatment (79.6%, *n* = 78), while 2.0% (*n* = 2) received combined therapy, and 18.4% (*n* = 18) had an unclear duration of treatment. Detailed information can be found in Table 3.

**Table 3:** Antiretroviral Treatment of pregnant Women living with HIV and neonates (*n* = 98).

Variable	*n*	%
Treatment of women living with HIV with any antiretroviral therapy		
Yes	65	66.3
No	33	33.7
The starting point of antiretroviral therapy for women living with HIV		
Before pregnancy	2	2
During pregnancy	58	59.2
At or after delivery	18	18.4
Unclear	20	20.4
Treatment strategies used for positive women		
Lamivudine/zidovudine/lopinavirritonavir	21	21.4
Lamivudine/tenofovir/lopinavirritonavir	28	28.6
Others	12	12.2
Unclear	37	37.8
Type of delivery		
Vaginal delivery	35	35.8
Cesarean section	60	61.2
Unclear	3	3
Type of feeding		
Breastfeeding	6	6
Formula feeding	93	94
Antiretroviral prophylaxis for neonates		
Yes	84	85.7
No	14	14.3
Preventive treatment strategies for Neonates		
Within 28 days	14	14.3
Between 29 days and 42 days	10	10.2
More than 42 days	57	58.2
Unclear	17	17.3
Duration of preventive treatment for neonates		
Monotherapy	78	79.6
Combined therapy	2	2
Unclear	18	18.4

### 3.4. Associations between characteristics and mother-to-child HIV transmission

Among 98 participants, age, marital status, and educational background showed no significant association. Similarly, the status of sexual partner, spousal therapy, and the number of children did not show significant associations. The gender of the child, planning for another pregnancy, and treatment of women with antiretroviral therapy had no significant impact. However, treatment strategies for positive women and the starting point of antiretroviral therapy for women living with HIV were associated with mother-to-child transmission (*P* < 0.05). Moreover, type of feeding, antiretroviral prophylaxis for neonates, and preventive treatment strategies for neonates were significantly associated with mother-to-child transmission. The preventive treatment program for neonates demonstrated no significant association. Detailed information can be found in Table 4.

**Table 4:** Factors associated with mother-to-child HIV transmission.

Variable	*N*		HIV-negative neonates (*N* = 88)	HIV-positive neonates (*N* = 10)	*χ*^2^ or corrected *χ*^2^	*P*-value (*P* < 0.05)
	Valid	Deficiencies				
Age (*n*, %)	98	0			0.285	1
20–29			28 (31.8)	3 (30)		
30–39			54 (61.4)	7 (70)		
≥40			6 (6.8)	0		
Marital status (*n*, %)	98	0				1
Unmarried			86 (97.7)	10 (100)		
Married			2 (2.3)	0		
Educational background (*n*, %)	98	0				0.349
Junior high school			74 (84.1)	10 (100)		
Senior high school			14 (15.9)	0		
Status of sexual partner (*n*, %)	98	0			0.591	0.442
Negative			55 (62.5)	5 (50)		
Positive			33 (37.5)	5 (50)		
Spousal therapy antiretroviral therapy (*n*, %)	98	0			0.028	0.867
Yes			28 (31.8)	4 (40)		
No			60 (68.2)	6 (60)		
Number of children (*n*, %)	98	0			1.741	0.38
1			56 (63.6)	8 (80)		
2			26 (29.5)	1 (10)		
3			6 (6.8)	1 (10)		
Gender of child (*n*, %)	85	13			0.138	0.71
Male			41 (46.6)	6 (60)		
Female			35 (39.8)	3 (30)		
Planning for another pregnancy (*n*, %)	98	0			2.027	0.155
Yes			28 (31.8)	6 (60)		
No			60 (68.2)	4 (40)		
Treatment of women living with HIV with any antiretroviral therapy (*n*, %)	98	0			4.893	0.027
Yes			62 (70.5)	3 (30)		
No			26 (29.5)	7 (70)		
The starting point of antiretroviral therapy for women living with HIV (*n*, %)	98	20			10.4	0.006
Before pregnancy			2 (2.3)	0		
During pregnancy			58 (65.9)	0		
After delivery			15 (17)	3 (30)		
Treatment strategies used for positive women (*n*, %)	61	37			3.637	0.133
Lamivudine/zidovudine/lopinavirritonavir			21 (23.9)	0		
Lamivudine/tenofovir/lopinavirritonavir			27 (30.7)	1 (10)		
Others			10 (11.4)	2 (20)		
Type of delivery (*n*, %)	95	3				1
Vaginal delivery			32 (36.4)	3 (30)		
Cesarean section			54 (61.4)	6 (60)		
Type of feeding (*n*, %)	98	0			29.286	0
Breastfeeding			1 (1.1)	5 (50)		
Formula feeding			87 (98.9)	5 (50)		
Antiretroviral prophylaxis for neonates (*n*, %)	98	0			15.076	0
Yes			80 (90.9)	4 (40)		
No			8 (9.1)	6 (60)		
Preventive treatment strategies for neonates (*n*, %)	81	17			1.328	0.516
Within 28 days			14 (15.9)	0		
Between 29 days and 42 days			9 (10.2)	1 (10)		
More than 42 days			54 (61.4)	3 (30)		
The preventive treatment program for neonates (*n*, %)	80	18				1
Combined therapy			74 (84.1)	4 (40)		
Monotherapy			2 (2.3)	0		

### 3.5. Binary logistic regression analysis

Binary logistic regression analyses were performed to determine the association between specific variables and mother-to-child HIV transmission. The results indicated that breastfeeding [B = 3.492, SE = 1.547, Wald = 5.097, *P* = 0.024, Exp(B) = 32.852, 95% CI = 1.585–680.99], antiretroviral prophylaxis for neonates [B = −0.914, SE = 1.288, Wald = 0.504, *P* = 0.478, Exp(B) = 0.401, 95% CI = 0.032–5.006], and treatment of women living with HIV with any antiretroviral therapy [B = −0.274, SE = 1.029, Wald = 0.071, *P* = 0.79, Exp(B) = 0.76, 95% CI = 0.101–5.715] were not significantly associated with mother-to-child HIV transmission. The constant term in the regression model was not statistically significant (B = −1.883, SE = 1.092, Wald). Detailed information can be found in Table 5.

**Table 5:** Binary logistic regression analyses.

	B	SE	Wald	df	*P*	Exp(B)	Exp(B) 95% CI	
							Lower	Upper
Breastfeeding	3.492	1.547	5.097	1	0.024	32.852	1.585	680.99
Antiretroviral prophylaxis for neonates	−0.914	1.288	0.504	1	0.478	0.401	0.032	5.006
Treatment of women living with HIV with any antiretroviral therapy	−0.274	1.029	0.071	1	0.79	0.76	0.101	5.715
Constants	−1.883	1.092	2.972	1	0.085	0.152		

## 4. Discussion

This study investigated the factors associated with mother-to-child HIV transmission and provided valuable insights into the demographic characteristics and interventions utilized in the prevention of transmission. The findings highlighted the critical role of healthcare providers in assessing HIV-infected mothers and their families to determine the appropriate feeding method, taking into account factors such as acceptability of artificial feeding, knowledge and skills, financial burden, access to safe milk substitutes, and the need for guidance and support. For eligible women, artificial feeding was recommended, while those choosing to breastfeed were provided with counseling and guidance to adhere to exclusive breastfeeding practices with continued antiviral treatment (17).

The study also revealed the migrant pregnant women, who predominantly belonged to the sexually active age group with low education and income levels. Lack of timely HIV treatment due to inadequate prenatal care and limited healthcare access emerged as a significant cause of mother-to-child transmission in this population (12). Strengthening primary healthcare, health insurance coverage, and maternal health knowledge is essential in reducing delays in testing and diagnosis and increasing opportunities for antiretroviral therapy (18).

Antiretroviral therapy was found to be a highly effective strategy in preventing mother-to-child HIV transmission, with a notable proportion of pregnant women receiving treatment. However, efforts are needed to improve access and utilization of antiretroviral therapy among HIV-positive pregnant women. The most common treatment regimens aligned with recommended guidelines, emphasizing the importance of standardized protocols for optimal outcomes.

Cesarean section was the predominant mode of delivery, aligning with established practices to minimize perinatal transmission. However, individualized circumstances and risks associated with each delivery method should be considered to ensure favorable outcomes for both mother and child. Formula feeding was prevalent among neonates, reflecting a cautious approach to reduce the risk of transmission through breastfeeding. However, comprehensive support and education are crucial to promote safe infant feeding practices, considering cultural, social, and individual factors influencing decision-making.

The relatively high coverage of antiretroviral prophylaxis for neonates was encouraging in terms of reducing vertical transmission risk. However, efforts should be directed towards addressing factors contributing to the proportion of neonates who did not receive prophylaxis to ensure that all eligible infants receive appropriate preventive measures.

The results of binary logistic regression analyses indicated that breastfeeding, antiretroviral prophylaxis for neonates, and treatment of women living with HIV were not significantly associated with mother-to-child HIV transmission. This suggests the involvement of other unexplored factors in influencing transmission outcomes. Future research should consider additional variables and potential confounders to gain a more comprehensive understanding of these factors.

To gain a broader perspective on the findings, we compared our results with other studies from China and other Asian countries. Thaker and Snow 1 conducted a study in a similar context, focusing on HIV viral suppression in the era of antiretroviral therapy. While their study provided insights into treatment outcomes, our study complements this by investigating the transmission dynamics among migrant populations. Y et al. (2) explored the process of preventing mother-to-child transmission of HIV, providing valuable guidance on prevention strategies. Our study's emphasis on antiretroviral prophylaxis for neonates aligns with their recommendations for preventive measures. Gong et al. (3) investigated the mother-to-child HIV transmission program in Suzhou, China, offering insights into regional differences and implementation challenges. Our study corroborates their findings and underscores the importance of tailored interventions for migrant populations. Zeng et al. (4) conducted a systematic review and meta-analysis of the prevention of mother-to-child HIV transmission cascade in China, which emphasized the need for comprehensive prevention programs. In addition, research from other Asian countries has also found significant effectiveness of preventive measures in reducing transmission risk. For instance, studies conducted in countries like Thailand, Malaysia, and the Philippines have also emphasized the importance of preventive measures (4, 11, 18). Our study aligns with their call for targeted interventions and highlights the risk associated with breastfeeding.

This study's comparative analysis deepens our understanding of mother-to-child HIV transmission among migrant populations in China and other Asian countries. It adds to existing knowledge on this critical public health issue and highlights the challenges faced by migrant pregnant women living with HIV. The insights gained can inform tailored intervention strategies to address their unique needs. The study emphasizes the importance of preventive measures and appropriate treatment strategies to reduce transmission risk. While antiretroviral prophylaxis for neonates shows promising coverage, identifying breastfeeding as a significant risk factor calls for targeted counseling and support for HIV-positive mothers considering breastfeeding.

## 5. Limitations

As a cross-sectional study, the findings provide associations rather than causality. The sample size of 98 participants might limit the generalizability of the results to a broader population. Additionally, recall bias and potential confounders may have influenced the results. Future studies with larger sample sizes and longitudinal designs are warranted to further explore the factors influencing mother-to-child HIV transmission in migrant populations.

## 6. Conclusion

This cross-sectional study sheds light on the mother-to-child transmission of HIV among migrant pregnant women and their neonates. The findings underscore the importance of tailored interventions to prevent transmission, including antiretroviral prophylaxis for neonates and appropriate treatment strategies for positive women. Addressing the risk associated with breastfeeding is crucial to improve the outcomes of HIV-exposed infants. The study contributes to the growing body of knowledge on HIV transmission in migrant populations and informs public health strategies for HIV prevention and management in this vulnerable group. Further research is needed to confirm and extend these findings for better maternal and child health outcomes.

## Data Availability

The original contributions presented in the study are included in the article/Supplementary Material, further inquiries can be directed to the corresponding author.
